# PrEP use and HIV seroconversion rates in adolescent girls and young women from Kenya and South Africa: the POWER demonstration project

**DOI:** 10.1002/jia2.25962

**Published:** 2022-07-13

**Authors:** Connie L. Celum, Elizabeth A. Bukusi, Linda Gail Bekker, Sinead Delany‐Moretlwe, Lara Kidoguchi, Victor Omollo, Elzette Rousseau, Danielle Travill, Jennifer F. Morton, Felix Mogaka, Gabrielle O'Malley, Gena Barnabee, Ariane van der Straten, Deborah Donnell, Urvi M. Parikh, Lauren Kudrick, Peter L. Anderson, Jessica E. Haberer, Linxuan Wu, Renee Heffron, Rachel Johnson, Susan Morrison, Jared M. Baeten, the POWER Study Team, Josephine Odoyo, Hilda Machafu, Mary‐Josephine Osore, Ethel Osome, Fredrick Omondi, Ben Kwach, David Ang'awa, Bernard Nyerere, Merceline Awuor, Annabel Dola, John Bosco Tsetso, Sherine Odek, Sylvia Mugalla, Vincent Momanyi, Peris Otieno, Brenda Misiko, Joel Odondi, Calvin Obuya, Boblief Otieno, Eric Seda, Alfred Obiero, Rachel Mwakisha, Petronilla Njenga, KMET staff, JOOTRH staff, Dorothy Zakariya, Lindile Nonjinge, Robin Julies, Pamela Fuzile, Thando Xeketwana, Lwazi Thami, Nkosiyabo Futshane, Desmond Raqa, Nomvuyiseko Mkatshana, Yolanda Mpanda, Weltevreden clinic staff, Thapelo Tlou, Kefilwe Kgabo, Samukelo Mbele, Phumzile Nyamane, Lerato Lunika, Sanele Gumede, Melt Ndlovu, Nomhle Khoza, Jeppestown clinic staff

**Affiliations:** ^1^ Department of Global Health University of Washington Seattle Washington USA; ^2^ Department of Medicine University of Washington Seattle Washington USA; ^3^ Department of Epidemiology University of Washington Seattle Washington USA; ^4^ Department of Obstetrics and Gynecology University of Washington Seattle Washington USA; ^5^ Center for Microbiology Research Kenya Medical Research Institute Kisumu Kenya; ^6^ Desmond Tutu HIV Centre Cape Town South Africa; ^7^ Wits RHI University of the Witwatersrand Johannesburg South Africa; ^8^ Center for AIDS Prevention Studies, Department of Medicine University of California San Francisco San Francisco California USA; ^9^ Fred Hutchinson Cancer Center Seattle Washington USA; ^10^ University of Pittsburgh Pittsburgh Pennsylvania USA; ^11^ University of Colorado‐Anschutz Medical Campus Aurora Colorado USA; ^12^ Center for Global Health Massachusetts General Hospital Boston Massachusetts USA; ^13^ Department of Medicine Harvard Medical School Boston Massachusetts USA; ^14^ Gilead Sciences Foster City California USA

**Keywords:** HIV, pre‐exposure prophylaxis, adherence, persistence, young women, Africa

## Abstract

**Introduction:**

HIV incidence remains high among African adolescent girls and young women (AGYW). The primary objective of this study is to assess pre‐exposure prophylaxis (PrEP) initiation, use, persistence and HIV acquisition among African AGYW offered PrEP in order to inform PrEP scale‐up.

**Methods:**

POWER was a prospective implementation science evaluation of PrEP delivery for sexually active HIV‐negative AGYW ages 16–25 in family planning clinics in Kisumu, Kenya and youth and primary healthcare clinics in Cape Town and Johannesburg, South Africa. Follow‐up visits occurred at month 1 and quarterly for up to 36 months. PrEP users were defined based on the month 1 refill. PrEP persistence through month 6 was assessed using Kaplan–Meier survival analysis among AGYW with a month 1 visit, defining non‐persistence as an ≥15 day gap in PrEP availability for daily dosing. PrEP execution was evaluated in a subset with PrEP supply from the prior visit sufficient for daily dosing by measuring blood tenofovir diphosphate (TFV‐DP) levels.

**Results:**

From June 2017 to September 2020, 2550 AGYW were enrolled (1000 in Kisumu, 787 in Cape Town and 763 in Johannesburg). Median age was 21 years, 66% had a sexual partner of unknown HIV status, and 29% had chlamydia and 10% gonorrhoea. Overall, 2397 (94%) initiated PrEP and 749 (31%) had a refill at 1 month. Of AGYW who could reach 6 months of post‐PrEP initiation follow‐up, 128/646 (20%) persisted with PrEP for 6 months and an additional 92/646 (14%) had a gap and restarted PrEP. TFV‐DP levels indicated that 47% (91/193) took an average of ≥4 doses/week. Sixteen HIV seroconversions were observed (incidence 2.2 per 100 person‐years, 95% CI 1.2, 3.5); 13 (81%) seroconverters either did not have PrEP dispensed in the study interval prior to seroconversion or TFV‐DP levels indicated <4 doses/week in the prior 6 weeks.

**Conclusions:**

In this study of PrEP integration with primary care and reproductive health services for African AGYW, demand for PrEP was high. Although PrEP use decreased in the first months, an important fraction used PrEP through 6 months.  Strategies are needed to simplify PrEP delivery, support adherence and offer long‐acting PrEP options to improve persistence and HIV protection.

## 1 INTRODUCTION

African adolescent girls and young women (AGYW) have had HIV incidence rates of 4% in recent HIV prevention research cohorts [1, 2, 3]. Oral tenofovir‐based pre‐exposure prophylaxis (PrEP) confers >90% protection against HIV when taken with high but not perfect adherence [4, 5, 6, 7]. Beginning in 2016 when Kenya and South Africa incorporated PrEP into HIV guidelines [8, 9, 10], PrEP access has increased for African AGYW.

Given the critical need for primary HIV prevention in this population, models of PrEP delivery are needed that reach AGYW and that foster PrEP use, specifically initiation, use (or execution) and persistence [11]. AGYW have trouble accessing reproductive healthcare due to transportation, long wait times, understaffed clinics and stigma from healthcare providers and community members about sexual activity among young, unmarried women [12, 13]. Family planning clinics, youth‐friendly clinics and mobile clinics provide sexually active youth with sexual and reproductive health (SRH) services, but little is known about whether AGYW will initiate and persist with PrEP in these settings.

The Prevention Options for Women Evaluation Research (POWER) study in Kenya and South Africa evaluated PrEP delivery models for African AGYW in diverse health settings, with the objective to assess PrEP initiation, use/execution and persistence when PrEP was offered as a routine service with minimal research procedures.

## 2 METHODS

### 2.1 Study population

From June 2017 through September 2020, AGYW in Kisumu, Kenya, and Cape Town and Johannesburg, South Africa were recruited (ClinicalTrials.gov NCT03490058) through peer outreach, social media and clinics. At enrolment, women were offered sexually transmitted infection (STI) testing and treatment, HIV testing, contraception and PrEP. Women were eligible if they were HIV negative by rapid tests, had vaginal intercourse in the prior 3 months and were interested in PrEP, PrEP initiation was not a requirement for enrolment. In Kisumu and Cape Town, women were eligible if they were between 16 and 25 years of age. In Johannesburg, if they were 18–25 years and urine pregnancy tests were negative.

The POWER study was implemented in facility‐based clinics and a mobile van providing reproductive health services to AGYW seeking family planning, SRH services and primary care, from which lessons about PrEP delivery could be adapted for programmatic scale up. Participating sites were family planning clinics in Kisumu (Kisumu Medical and Education Trust, KMET, and Jaramogi Oginga, Odinga Teaching and Referral Hospital, JOOTRH), the Tutu Teen Truck in townships near Cape Town [14], the Weltevreden public primary healthcare clinic in Cape Town and a youth‐friendly primary healthcare clinic (Ward 21) and public primary healthcare clinic (Jeppestown) in Johannesburg.

### 2.2 Study procedures

Women were counselled about risk reduction and HIV prevention and given the option to initiate PrEP. Women could decline PrEP or opt to stop PrEP at any time, and those who stopped PrEP could restart. Follow‐up occurred at 1 month after enrolment (or when PrEP was initiated, if after enrolment) and then quarterly for up to 36 months. Women were reminded of visits through text messages, WhatsApp messages or phone calls. Women were not provided travel reimbursement or incentives for participation and field‐based retention activities were not conducted, as programmatic HIV prevention settings do not provide compensation or active outreach.

Data collected at enrolment were limited to what is obtained in PrEP delivery programs, which did not include age of male partners. Enrolment data were used to calculate the modified VOICE HIV risk score with a maximum possible modified VOICE risk score of 8; a VOICE score of 5 or higher was associated with an annual HIV incidence of 6% in prior cohorts [15].

#### 2.2.1 Laboratory testing

Rapid HIV testing was performed at PrEP initiation and follow‐up visits with reactive tests confirmed by local laboratories. HIV resistance testing on samples from seroconverters was performed using a commercial (ThermoFisher Scientific) or validated HIV‐1 population genotyping assay with a partial reverse transcriptase gene region [16]. *Chlamydia trachomatis* and *Neisseria gonorrhoeae* nucleic acid amplification testing was performed by local laboratories at enrolment and 6 months. At PrEP initiation, hepatitis B surface antigen and creatinine clearance was measured using the Cockcroft–Gault equation. Urine pregnancy testing was performed when clinically indicated in the Kenyan sites and at all PrEP dispensation visits in the South African sites, following national guidelines.

PrEP was withheld from women who had a reactive rapid HIV test, decreased creatinine clearance (<50 ml/minute in Kenya and <60 ml/minute in South Africa) and for South African participants who became pregnant, consistent with national guidelines at the time. Women could restart PrEP if confirmatory testing showed they were HIV negative, their estimated creatinine clearance returned to normal or after pregnancy. Women could take PrEP while breastfeeding.

Dried blood spots (DBS) for measurement of intracellular tenofovir diphosphate (TFV‐DP) were collected at enrolment and all follow‐up visits, but due to women's dislike of phlebotomy was changed to the 6 months visit after PrEP initiation when creatinine was measured to avoid an additional blood draw. Participants who were not comfortable with phlebotomy could decline DBS. DBS samples were obtained from HIV seroconverters at the first HIV‐positive visit. Intracellular TFV‐DP levels provide a measure of average PrEP adherence in the prior 6 weeks [17]. TFV‐DP levels were quantified at the University of Colorado, Denver, using an updated validated extraction procedure [18]. DBS thresholds were established from directly observed FTC‐TDF dosing; TFV‐DP levels of ≥1450 fmol/punch associated with seven doses per week, 800–1449 fmol/punch with 4–6 doses per week, 400–799 fmol/punch with 2–3 doses/per week and <400 for fmol/punch with <2 doses per week.

For seroconverters who did not have a DBS sample, plasma tenofovir levels were measured using a validated ultra‐performance liquid chromatographic, tandem mass spectrometry assay with linear values from 0.5 to 500 ng/ml, and lower limit of quantification of 0.5 ng/ml. Plasma TFV >40 ng/ml was correlated with 90% efficacy in the Partners PrEP Study [19].

### 2.3 Ethical review

The study was approved by ethics review committees at the Kenya Medical Research Institute, JOOTRH Independent Ethics Review Committee, University of Cape Town, University of Witwatersrand and University of Washington. Participants provided written informed consent in English or local languages. Parental consent was waived for participants ages 16–17 years in Kisumu and Cape Town.

### 2.4 Statistical analysis

The goal was to enrol 500 participants per site and up to 1000 participants per country, providing diversity by site and country with respect to PrEP delivery. Formal sample size calculations were not conducted. Descriptive analyses assessed participant demographic and behavioural characteristics by site. PrEP initiation was defined as receiving a bottle with 30 tablets at enrolment and PrEP use at 1 month as attending a visit 1 month post‐PrEP initiation and receiving a 90 day refill. PrEP persistence was assessed among women receiving a PrEP refill at 1 month; non‐persistence was defined as a gap of ≥15 days between the refill return date and the next PrEP dispensation (assuming daily dosing prior to the next PrEP dispensation). Six months is a typical period for evaluation of PrEP adherence and persistence in PrEP demonstration projects among African AGYW who have low risk perception and dynamic partnerships. PrEP persistence was defined as persistent use through 6 months and PrEP refill at the 6 month visit. The cumulative probability of PrEP persistence during the 6 months after PrEP initiation was modelled using the Kaplan–Meier estimator of the survival function. Analysis of the predictors of PrEP persistence beyond 6 months used multivariable Poisson regression, including variables significant at a *p*‐value<0.2 in the univariate analysis. The modified VOICE risk score was included as a categorical variable operationalized as 0–4, 5–6, 7–8 or missing.

PrEP availability was estimated for maximal coverage as the doses that could have been taken given the number of PrEP refills dispensed and the number of days of follow‐up. For PrEP execution, intracellular TFV‐DP was assessed from visits 3 or 6 months after enrolment. A subset of 11% samples were randomly selected from the two sites, which sent all DBS samples collected were tested, as well as all 33% samples sent from the third site.

All descriptive statistics and the Kaplan–Meier curve were generated in SAS 9.4 (SAS/STAT 14.1, Cary, NC, USA). Regression models for PrEP persistence were generated using R version 4.0.3.

## 3 RESULTS

### 3.1 Study participants

Between June 2017 and September 2020, 2550 AGYW were enrolled: 1000 from Kisumu, 787 from Cape Town and 763 from Johannesburg (Table 1). Their median age was 21 years (interquartile range, IQR 19–23), and all had a current partner, 66% of whom AGYW were reported to have unknown HIV status and 4% to be living with HIV. Seventy‐eight percent reported not knowing if their partner had other partners and 22% that their partner had other partners. Almost half (47%) were using contraception at enrolment, of whom 8% used oral contraception, 51% injectable contraception (depot medroxyprogesterone acetate or norethisterone enantate) and 27% a contraceptive implant. At enrolment, although only 7% reported STI symptoms, 29% were diagnosed with *C. trachomatis* and 10% with *N. gonorrhoeae*. Among those diagnosed with *C. trachomatis* or *N. gonorrhoeae*, STI symptoms were reported by 23% (179/770).

**Table 1:** Baseline demographic and behavioural characteristics of POWER participants

		Kenya		South Africa			
		Kisumu		Cape Town		Johannesburg	
Characteristic	Total (*n* = 2550)	JOOTRH (*n* = 496)	KMET (*n* = 504)	TTT (*n* = 681)	Weltevreden (*n* = 106)	Jeppestown (*n* = 399)	Ward 21 (*n* = 364)
Age (years), median (IQR)	21 (19–23)	21 (19–23)	21 (19–23)	20 (18–22)	19.5 (18–22)	21 (20–23)	21 (19–22.5)
Marital status^a^							
Single, no partner	40 (2%)	4 (<1%)	10 (2%)	12 (2%)	4 (4%)	4 (1%)	6 (2%)
Single, has partner	2154 (85%)	312 (63%)	355 (70%)	661 (97%)	101 (95%)	381 (96%)	344 (95%)
Married; husband's only wife	330 (13%)	167 (34%)	130 (26%)	7 (1%)	1 (<1%)	12 (3%)	13 (4%)
Sexual partners, past 3 months							
Current *N*, median (IQR)	1 (1–1)	1 (1–1)	1 (1–2)	1 (1–1)	1 (1–1)	1 (1–1)	1 (1–1)
HIV‐positive sex partner	106 (4%)	35 (7%)	14 (3%)	26 (4%)	3 (3%)	14 (4%)	14 (4%)
Sex partner, unknown HIV status	1672 (66%)	302 (61%)	305 (61%)	555 (82%)	90 (85%)	227 (57%)	193 (53%)
Knows or thinks partner has other partners	549 (22%)	90 (18%)	178 (36%)	134 (20%)	14 (13%)	68 (17%)	65 (18%)
Condom use							
Never	689 (27%)	219 (44%)	166 (33%)	137 (20%)	28 (26%)	97 (24%)	42 (12%)
Sometimes	1509 (59%)	233 (47%)	285 (57%)	449 (66%)	60 (57%)	239 (60%)	243 (67%)
Always	341 (13%)	42 (9%)	49 (10%)	91 (13%)	18 (17%)	62 (16%)	79 (22%)
Any alcohol use in the past 3 months	1056 (41%)	38 (8%)	69 (14%)	514 (76%)	78 (74%)	173 (43%)	184 (51%)
Drinks per week, median (IQR)	3 (1–5)	1 (1–1)	1 (1–2.5)	4 (2–5)	5 (4–5)	2 (1–5)	1.5 (1–4)
STI diagnosis							
*Chlamydia trachomatis*	667 (29%)	77 (16%)	95 (19%)	259 (41%)	55 (52%)	113 (35%)	68 (27%)
*Neisseria gonorrhoea*	221 (10%)	26 (5%)	35 (7%)	100 (16%)	21 (20%)	25 (8%)	14 (6%)
STI symptoms	179 (7%)	37 (7%)	82 (16%)	27 (4%)	6 (6%)	8 (2%)	19 (5%)
HIV risk							
Modified VOICE risk score^b^; median (IQR)	6 (5–7)	6 (4–6)	6 (4–6)	7 (6–8)	7 (7–8)	6 (5–7)	6 (5–7)
Contraceptive use							
Any	1208 (47%)	181 (36%)	248 (49%)	324 (48%)	56 (53%)	192 (48%)	207 (57%)
Oral	92 (8%)	4 (2%)	8 (3%)	11 (3%)	2 (4%)	37 (19%)	30 (15%)
Injectable	604 (51%)	34 (19%)	66 (27%)	248 (77%)	45 (80%)	97 (51%)	114 (58%)
Implant	317 (27%)	86 (48%)	140 (57%)	44 (14%)	8 (14%)	26 (14%)	13 (7%)
IUD	34 (3%)	12 (7%)	8 (3%)	6 (2%)	0 (0%)	1 (<1%)	7 (4%)
Condoms	191 (16%)	45 (25%)	25 (10%)	13 (4%)	1 (2%)	28 (15%)	79 (40%)
Ever pregnant	1213 (48%)	238 (48%)	291 (58%)	221 (33%)	29 (27%)	276 (70%)	158 (43%)
Currently pregnant	162 (6%)	129 (26%)	31 (6%)	2 (0%)	0 (0%)	0 (0%)	0 (0%)
Laboratory screening for PrEP							
Creatinine clearance ml/minute, median (IQR)	135 (110–164)	130 (111–154)	97 (83–113)	152 (128–179)	152 (126–183)	144 (122–176)	143 (122–174)
Creatinine clearance >60 ml/minute	2344 (99%)	427 (100%)	429 (97%)	643 (100%)	103 (100%)	380 (100%)	362 (100%)
Hepatitis B surface Ag positive	14 (<1%)	5 (1%)	4 (<1%)	3 (<1%)	1 (<1%)	0	1 (<1%)
Initiated PrEP^c^	2397 (94%)	440 (89%)	459 (91%)	659 (97%)	103 (97%)	379 (95%)	357 (98%)

^a^ <1% of women were in a polygamous marriage, widowed or divorced.

^b^ The modified VOICE risk score includes age<25; not married or living with partner; alcohol use in the past 3 months; partner does not provide financial/material support; and partner has other sex partners with a maximum score of 8.

^c^ 2359 participants initiated PrEP at enrolment and 38 after enrolment.

### 3.2 PrEP initiation, use/execution and persistence

**Figure 1:**
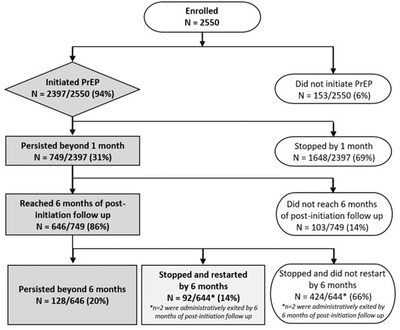
PrEP initiation and persistence beyond 6 months among women who initiated PrEP. The flow chart depicts PrEP initiation and persistence among the 2550 women who enrolled in the POWER cohort. PrEP persistence was assessed based on attendance of the 1 month visit for a PrEP refill, and persistence through 6 months among those reached at least 6 months of post‐PrEP initiation follow‐up time. PrEP non‐persistence was defined as a gap of ≥15 days between the refill return date and the next PrEP refill dispensation, assuming daily dosing prior to the next PrEP dispensation date. PrEP persistence beyond 6 months was defined as persistent use through 6 months and PrEP refill at the visit 6 month visit.

Over 99% of women were eligible for same day PrEP initiation based on an estimated creatinine clearance >60% (or >50% in Kenya) and <1% had a positive hepatitis B surface antigen. Of the 2550 women enrolled, 2397 (94%) initiated PrEP, 749 (31%) returned at 1 month and received a refill (Figure 1). Information is not available about the reported reasons for the 69% who did not return at 1 month. Of the 646 who returned at month 1 and had 6 or more months of follow‐up time to assess PrEP persistence over 6 months, 20% persisted with PrEP to 6 months without an ≥15 day gap in refills, and 14% restarted PrEP after a gap of ≥15 days (Figure 1).

**Figure 2:**
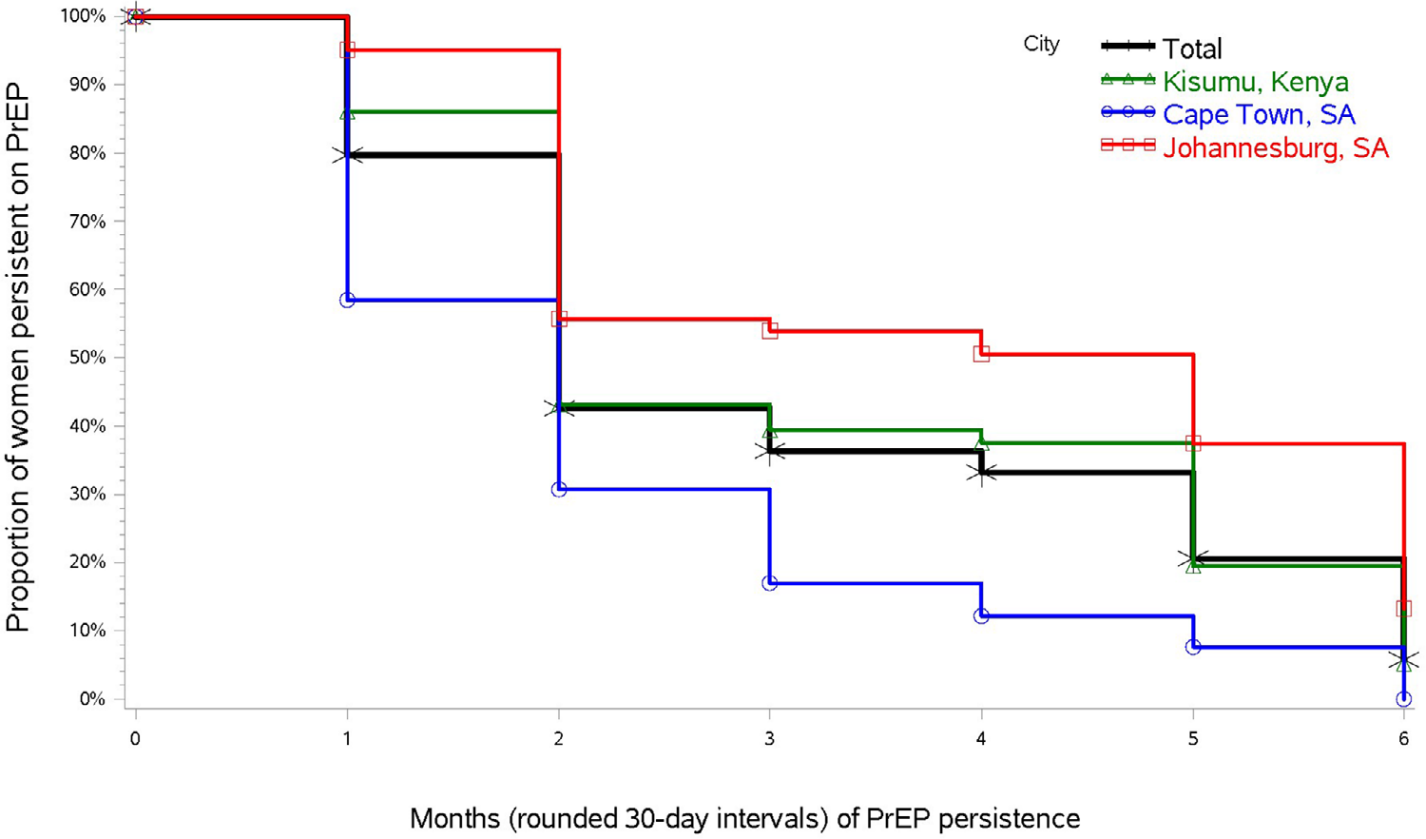
PrEP persistence among women who had PrEP persistence beyond 1 month. The cumulative probability of a PrEP non‐persistence failure event within 6 months of PrEP initiation was modeled using the Kaplan–Meier estimator of the survival function. The 749 women who had a PrEP refill at 1 month are included in the analysis. The vertical drop at each point indicates PrEP non‐persistence “events,” defined as a gap of ≥15 days between refills that would provide sufficient PrEP supply for daily dosing.

PrEP persistence beyond 6 months varied by site, ranging from 8% to 38% (Figure 2).

Older age, injectable contraception, intrauterine device or other contraceptive use, and reporting sometimes or always using condoms were significant baseline predictors of PrEP persistence beyond 1 month but not 6 months (Tables 2 and 3). The modified VOICE risk score was not predictive of PrEP persistence at 1 or 6 months.

**Table 2:** Baseline predictors of PrEP persistence beyond 1 month

	*N* (%) or median (IQR)		Univariate		Multivariate	
	PrEP persisters	PrEP non‐persisters	Relative risk (95% CI)	*p*‐value	Relative risk^a^ (95% CI)	*p*‐value
N participants	749 (31.3%)	1648 (68.8%)				
Age (years)	21 (19–23)	20 (19–23)	1.03 (1.01–1.06)	0.007	1.04 (1.01–1.07)	0.005
Contraceptive use						
None/condoms only	378 (27.5%)	999 (72.6%)	ref	0.135	ref	0.038
Oral	31 (34.4%)	59 (65.6%)	1.25 (0.93–1.69)	<0.001	1.37 (1.02–1.84)	<0.001
Injectable	214 (37.0%)	365 (63.0%)	1.35 (1.17–1.54)	0.022	1.37 (1.19–1.58)	0.066
Implant	101 (33.9%)	197 (66.1%)	1.23 (1.03–1.48)	<0.001	1.19 (0.99–1.44)	<0.001
IUD/other	25 (48.1%)	27 (51.9%)	1.75 (1.30–2.35)	0.135	1.65 (1.23–2.22)	0.038
Condom use, past 3 months						
None	179 (28.7%)	444 (71.3%)	ref	0.156	ref	
Sometimes	461 (31.9%)	984 (68.1%)	1.11 (0.96–1.28)	0.203	1.22 (1.05–1.43)	0.010
Always	105 (32.7%)	216 (67.3%)	1.14 (0.93–1.39)		1.26 (1.02–1.55)	0.031
Modified VOICE risk score^a^, ^b^						
0–4	164 (35.5%)	298 (64.5%)	ref			
5–6	273 (30.3%)	629 (69.7%)	0.85 (0.73–1.00)	0.048	0.88 (0.75–1.05)	0.149
7–8	305 (30.0%)	709 (69.9%)	0.85 (0.73–0.99)	0.036	0.87 (0.73–1.05)	0.144

^a^ Adjusted for site.

^b^ Maximum score on the modified VOICE risk score is eight.

**Table 3:** Baseline predictors of PrEP persistence beyond 6 months, among women who persisted beyond 1 month

	*N* (%) or median (IQR)		Univariate		Multivariate	
	PrEP persisters	PrEP non‐persisters	Relative risk (95% CI)	*p*‐value	Relative risk^a^ (95% CI)	*p*‐value
N participants	128 (19.8%)	518 (80.2%)				
Age (years)	21 (20–23)	21 (19–23)	1.05 (0.99–1.13)	0.123	1.01 (0.94–1.09)	0.729
Contraceptive use						
None/condoms only	57 (17.5%)	268 (82.5%)	ref		ref	
Oral	1 (44.4%)	15 (55.6%)	2.53 (1.56–4.12)	<0.001	1.84 (1.11–3.05)	0.018
Injectable	39 (21.7%)	141 (78.3%)	1.24 (0.86–1.78)	0.255	1.23 (0.86–1.74)	0.260
Implant	13 (14.1%)	79 (85.9%)	0.81 (0.46–1.41)	0.446	0.81 (0.46–1.41)	0.455
IUD/other	7 (31.8%)	15 (68.2%)	1.81 (0.94–3.49)	0.075	1.41 (0.74–2.68)	0.290
Condom use, past 3 months						
None	26 (16.6%)	131 (83.4%)	ref		–	–
Sometimes	84 (20.9%)	318 (79.1%)	1.26 (0.85–1.88)	0.254		
Always	18 (21.4%)	66 (78.6%)	1.29 (0.75–2.22)	0.349		
Modified VOICE risk score^a^, ^b^						
0–4	33 (22.5%)	114 (77.6%)	ref		ref	
5–6	57 (23.0%)	191 (77.0%)	1.02 (0.70–1.49)	0.903	1.04 (0.72–1.51)	0.818
7–8	38 (15.5%)	207 (84.5%)	0.69 (0.45–1.05)	0.084	0.82 (0.54–1.26)	0.369

^a^ Adjusted for site.

^b^ Maximum score on the modified VOICE risk score is 8.

Coverage calculations showed that 20% of women had sufficient PrEP supply to enable dosing ≥ 4 tablets per week over 6 months (data not shown). Among 1156 eligible for 3 and 6 month visits, 193 (16.7%) specimens were selected to measure intracellular TFV‐DP levels, which indicated that 19.0% had taken PrEP daily, 28.2% an average of 4–6 doses per week, 5.1% an average of 2–3 doses per week, 19.5% an average of 1 dose per week and 28.2% had TFV‐DP levels below the limit of quantification (BLQ, <31.25 fmol/punch; Table 4).

**Table 4:** PrEP adherence based on intracellular tenofovir diphosphate levels at month 3 or 6

Description	Total	JOOTRH and KMET Kisumu	Tutu Teen Truck Cape Town^a^	Ward 21 Johannesburg^a^
N samples tested (% of eligible visits^b^)	193 (16.7%)	65 (11.3%)	32 (11.1%)	96 (32.9%)
DBS TFV‐DP fmol/punch Median (IQR)	510 (16–1263)	BLQ^c^ (16–341)	770 (56–1404)	1063 (218–1394)
Estimated average weekly PrEP use^d^				
7 doses/week (≥1450 fmol/punch)	19.0%	10.8%	25.0%	21.9%
4–6 doses/week (800–1449 fmol/punch)	28.2%	9.2%	25.0%	41.7%
2–3 doses/week (400–799 fmol/punch)	5.1%	4.6%	3.1%	6.3%
<2 doses/week (31.25–399 fmol/punch)	19.5%	23.1%	28.1%	14.6%
None (BLQ^c^, <31.25 fmol/punch)	28.2%	52.3%	18.8%	15.6%

^a^ The three cities included two facilities each, two of which (Jeppestown clinic in Johannesburg and Weltevreden clinic in Cape Town) were excluded in this table because they had few or no specimens available for testing.

^b^ Eligible visits for TFV‐DP assessment were month 3 and/or 6 visits, where the participant had PrEP supply from the prior visit sufficient for daily dosing, from 11% of visits from participants with PrEP supply estimated to be sufficient for daily dosing were randomly selected from the two sites, which sent all DBS samples collected, and all from the third site, which sent a subset of 33% DBS samples collected.

^c^ Below limit of quantification (<31.25 fmol/punch).

^d^ Thresholds for intracellular tenofovir diphosphate levels in red blood cells associated with average doses per week in the 6 weeks prior to the DBS sample were based on directly observed dosing studies in studies of MSM, which measured TFV‐DP levels

### 3.3 HIV and STI incidence

Sixteen post‐PrEP initiation seroconversions were observed over 741.8 person‐years of follow‐up for an HIV incidence of 2.2 per 100 person‐years (95% confidence interval [CI] 1.2, 3.5). Four seroconversions were detected at month 1, potentially indicating that they were infected at PrEP initiation (Table S1). Seven of 12 (58%) women who seroconverted after month 1 were known to not be taking PrEP at the time of seroconversion either because of missing the prior PrEP refill, declining PrEP or having previously discontinued PrEP. Thirteen seroconverters had DBS samples available for intracellular TFV‐DP levels, and two had plasma tenofovir tested because DBS was not available. All four who seroconverted to HIV at month 1 had detectable TFV‐DP, two at levels consistent with taking an average of 4 or more times per week. For the remaining nine who seroconverted after month 1 and had DBS available, TFV‐DP was undetectable for six, two had levels consistent with taking less than 4 times/week and one had levels consistent with taking an average of 4 or more times per week. Plasma tenofovir levels for two seroconverters for whom DBS were not available had results of 79.1 ng/ml and undetectable (<0.5 ng/ml), respectively.

Fourteen seroconverters had samples from which HIV could be amplified for testing for antiretroviral resistance, two had M184M/V mutations and both had high TFV‐DP levels at 1 month. Ten had no resistance mutations and three had NNRTI mutations.

The incidence of *C. trachomatis* and *N. gonorrhoeae* was 42.9/100 person‐years (95% CI 37.2, 49.2)—35.4/100 person‐years for *C. trachomatis* (95% CI 30.3, 41.2) and 13.0/100 person‐years for *N. gonorrhoeae* (95% CI 10,0, 16.7) of whom 42% of the women also had *C. trachomatis* and/or *N. gonorrhoeae* at enrolment.

## 4 DISCUSSION

PrEP was offered at SRH and primary healthcare clinics for AGYW in Kenya and South Africa. Initiation of PrEP was very high (94%) among these sexually active AGYW who were seeking health services, and who had multiple risk factors for HIV, including inconsistent condom use, a partner of unknown HIV status and a curable STI. The high rate of PrEP initiation indicates that AGYW were motivated even though only one‐third returned for a PrEP refill at 1 month. Of those who returned at 1 month, one‐third persisted with PrEP through 6 months or restarted PrEP within 6 months after a gap of ≥15 days between PrEP refills. The high frequency of early drop‐off could reflect the lack of reimbursement for transport and study participation, or other challenges with continuing PrEP. Site differences in PrEP persistence may reflect differences in PrEP delivery strategies and regional differences in PrEP rollout and awareness. Although there was high early drop‐off, a minority of women were able to persist with PrEP for 6 months.

Due to this study having minimal research procedures, we are limited in understanding factors associated with women not returning for their first PrEP refill. Predictors of PrEP use at 1 month indicate factors that could identify AGYW who need more support for persisting with PrEP, including younger age, lack of condom use and oral or injectable hormonal contraception use. The only significant predictor of PrEP persistence beyond both 1 and 6 months was oral contraceptive use at baseline. Oral contraceptive pills are not typically recommended for African AGYW due to high pregnancy rates of up to 16% [20]. Our finding suggests that experience in daily pill‐taking for contraception facilitated taking pills consistently for HIV prevention, and supports the evaluation of a dual prevention tablet with oral contraception and PrEP [21].

The moderate level of PrEP persistence may be indicative of PrEP use in programmatic delivery without reimbursement or active retention efforts, particularly among young women with dynamic sexual relationships and for whom medication adherence is typically low [22, 23], who often have challenges recognizing their risk for HIV or maintaining motivation to attend clinic or daily pill‐taking [24]. Qualitative findings from this study indicate that PrEP use was hampered by low community awareness, stigma and misconceptions about PrEP, daily pill‐taking was challenging for AGYW and that PrEP disclosure, social support, adolescent‐friendly health counselling and convenient access were key enablers for PrEP persistence [25]. PrEP demonstration projects in Kenya and South Africa have identified the need for an enabling environment to reduce stigma about sexuality and support AGYW PrEP users [26, 27].

Short gaps in PrEP refills in one‐fifth of the AGYW who continued PrEP beyond 6 months indicate the frequency of PrEP “pauses” when providers need to reassess women's risk and counsel about restarting PrEP. Proactive call‐back mechanisms and express visits may aid women in continuing both PrEP and contraception given low contraceptive continuation rates in African settings [28] and that contraceptive use was a predictor of PrEP persistence in this study. The MTN 034/REACH cross‐over study of oral PrEP and the dapirivine ring in African AGYW demonstrated that in the context of being provided options for adherence support strategies and monthly visits, almost all had high adherence over 18 months [29]. These strategies should be adapted and evaluated in programmatic settings.

In this study and recent PrEP studies in African AGYW [30, 31], >99% were eligible for same day PrEP initiation based on normal creatinine clearance and lack of active hepatitis B infection. Laboratory screening should not be a barrier to PrEP delivery for young women. Health service barriers, including phlebotomy for laboratory testing, limited clinic hours, stock outs of commodities, including contraceptives, staffing and fast track queues, need to be addressed to facilitate PrEP delivery. These factors could also be challenged with PrEP dispensation and persistence for longer‐acting agents, such as the dapivirine ring and injectable cabotegravir. Innovative, community‐based PrEP delivery strategies are needed to reduce barriers, including transportation costs and inconvenience of clinic visits.

Consistent condom use was low and prevalence of *C. trachomatis* and *N. gonorrhoeae* was high, among whom only a small minority reported symptoms with annual incidence almost as high as their prevalence. Syndromic STI management would miss treatment of these asymptomatic, curable STIs. Similarly, high rates of *C. trachomatis* and *N. gonorrhoeae* were observed in recent PrEP demonstration projects [30, 31, 32], highlighting the need for integration SRH services and PrEP delivery for African AGYW, including etiologic STI diagnosis, prompt treatment and expedited partner treatment [33].

Two women who were identified as HIV seroconverters 1 month after PrEP initiation had PrEP‐associated resistance, specifically M184M/V mutations conferring resistance to emtricitabine. Both participants could have been infected at enrolment and not detected by standard antibody tests during HIV seroconversion when viral loads are often high. PrEP‐associated resistance mutations during undiagnosed acute HIV infection are rare [6, 34] and are balanced by the benefits of preventing HIV infection in the great majority of users [35].

HIV incidence in this cohort was 2.2 per 100 woman‐years, which is a potential overestimate since several infections were identified in the first 1–3 months after PrEP initiation could represent baseline infections, which could not be retrospectively assessed with more sensitive assays because samples were not archived at enrolment. The HIV incidence is higher than recent demonstration projects of PrEP in African AGYW [30, 31]; in this cohort, women who acquired HIV had stopped PrEP or had low use based on their TFV‐DP levels. A control group was not included for ethical reasons of withholding a proven efficacious prevention method (PrEP) to women at risk. Thus, the HIV incidence cannot be compared to a contemporaneous cohort of women who were not offered PrEP, although risk behaviours and STI prevalence are similar to recent cohorts of African AGYW with an HIV incidence of 4% [1, 2, 3]. Proactive outreach may be useful in the first few weeks after PrEP initiation in order to assess women's risk perception and PrEP motivations, challenges with PrEP use to mitigate early drop‐off, and provide adherence support and visit reminders to women at higher risk. These strategies may improve continuation of longer‐acting PrEP formulations, as they are implemented.

This study has the strengths of evaluating PrEP uptake and persistence among African AGYW attending diverse facility‐based clinics and mobile clinical services with minimal research procedures and no compensation for travel and visits. Thus, the observed rates of PrEP uptake and persistence are indicative of what may be observed in programmatic PrEP delivery. The study has limited information about barriers to PrEP use, persistence and reasons for not returning or discontinuing PrEP, and HIV infection prior to PrEP initiation was not definable for four women because enrolment samples were not archived. Quantification of adherence among seroconverters was limited due to the single time point for DBS or plasma collection.

## 5 CONCLUSIONS

While the high PrEP initiation rates among African AGYW and feasibility of integrating PrEP into SRH and primary care are encouraging, the findings highlight the need for simpler delivery through fast track visits and community‐based refills to better support PrEP persistence. Differentiated PrEP support strategies are needed when delivering PrEP at scale for AGYW to reduce early drop‐off after PrEP initiation. While less user‐dependent and longer‐acting PrEP formulations for example dapivirine ring and injectable cabotegravir, provide preferable options for some women, they will also need to be integrated into primary care and SRH services with regular interactions for HIV testing and safety monitoring.

## COMPETING INTERESTS

PLA reports grants from NIH, during the conduct of the study; grants and consulting fees from Gilead Sciences and advisory board fees from Merck, ViiV outside the submitted work; JMB reports grants from NIH during the conduct of the study; personal fees from Gilead Sciences, Janssen, Merck, outside the submitted work; CC reports grants from NIH and has received advisory board fees from Merck and Gilead Sciences outside the submitted work; DD reports grants from NIH; RH has received funding from NIH and Children's Investment Fund Foundation and consulting fees from World Health Organization outside the submitted work; JEH has received advisory board fees from Merck outside the submitted work; GOM received grants from CDC and NIH; UMP received grants from NIH, IPM and received personal fees from Merck outside the submitted work; ER received personal fees from Gilead Science outside the submitted work; and AvdS received personal fees from Merck outside the submitted work.

## AUTHORS’ CONTRIBUTIONS

CC, JMB and RJ contributed to funding acquisition. CC, JMB, DD, EB, SDM, LGB, GOM, AvdS, UMP, PLA, JEH and RH designed the study. RJ, VO, ER, DT, FM, GB, SM and JFM were responsible for project administration. LK and LW analysed the data. CC wrote the original draft. JMB, DD, LGB, EB, SDM, SM, GB, RJ, VO, ER, DT, FM, JFM, GB, GOM, AvdS, UMP, PLA, JEH and RH contributed to writing, reviewing and editing the manuscript.

## FUNDING

This study was funded by the United States Agency for International Development, Cooperative Agreement AID‐OAA‐A‐15‐00034.

## DISCLAIMER

Gilead Sciences donated tenofovir disoproxil fumarate‐emtricitabine to the study.

## Supporting information


**Table S1**. Summary of post‐PrEP seroconversions with PrEP refill history, initial HIV‐1 viral load and resistance mutations.

## Data Availability

Data may be available upon request.
