# Supplementary material for: PrEP use and HIV seroconversion rates in adolescent girls and young women from Kenya and South Africa: the POWER demonstration project
Source: J Int AIDS Soc. 2022 Jul 13;25(7):e25962. doi: 10.1002/jia2.25962 (PMC9278271; doi:10.1002/jia2.25962)
Supplement: Supplementary file 1 — Table S1. Summary of post‐PrEP seroconversions with PrEP refill history, initial HIV‐1 viral load and resistance mutations. [file JIA2-25-e25962-s001.docx]

**Appendix Table 1. Summary of post-PrEP seroconversions with PrEP refill history, initial HIV-1 viral load and resistance mutations**

| **Seroconverter #** | **Month after PrEP initiation** | **PrEP history,**  **by study visit** | **Viral load (copies/mL)** | **TFV-DP DBS or plasma TFV at SC visit** | **Resistance mutations** |
| --- | --- | --- | --- | --- | --- |
| 1 | 10 months | M0-M6: Accepted PrEP | 1020000 | DBS TFV level = BLQ | None |
| 2 | 8 months | M0-M1: Accepted PrEP  M3: PrEP hold | 42725 | DBS TFV level = 127 fmol/punch | E138A, G190A |
| 3 | 9 months | M0: Accepted PrEP M1: Accepted PrEP  M3: Declined PrEP  M6: Missed visit | 3100 | Plasma TFV level = BLQ | None |
| 4 | 3 months | M0: Accepted PrEP  M1: Missed visit | 39600 | DBS TFV level = BLQ | K103N |
| 5 | 8 months | M0-M3:Accepted PrEP | 50200 | Plasma TFV level = 79.1 ng/mL | None |
| 6 | 1 month | M0: Accepted PrEP | 1270000 | DBS TFV level = 85.5 | None |
| 7 | 23 months | M0: Accepted PrEP  M1: Missed visit  M3: Declined PrEP  M6-18: Missed visits | 5387 | DBS TFV level = BLQ | None |
| 8 | 19 months | M0: Accepted PrEP  M1-15: Missed visits | Declined blood draw | Declined blood draw | Declined blood draw |
| 9 | 1 month | M0: Accepted PrEP | 10827 | DBS TFV level = 998 fmol/punch | **M184V, M184MV**, K103N |
| 10 | 3 months | M0: Accepted PrEP  M1: Missed visit | 4291 | DBS TFV level = BLQ | None |
| 11 | 20 months | M0-1: Accepted PrEP  M3: Missed visit  M6: Accepted PrEP  M9-15 Missed visits | 8767 | DBS TFV level = BLQ | None |
| 12 | 1 month | M0: Accepted PrEP | <40 | DBS TFV level = 644 fmol/punch | Unable to amplify |
| 13 | 3 months | M0: Accepted PrEP  M1: Accepted PrEP | 143 | DBS TFV level = 832 fmol/punch | None |
| 14 | 21 months | M0, 1, 3, 6, 9, 12, 15, 18: Accepted PrEP | 3315 | DBS TFV level = 499 fmol/punch | Unable to amplify |
| 15 | 1 month | M0: Accepted PrEP | 11458 | DBS TFV level = 1247 fmol/punch | **M184MIV** |
| 16 | 6 months | M0: Accepted PrEP  M1: Missed visit  M3: Accepted PrEP | 487485 | DBS TFV level = BLQ | A98G |

**Appendix Table 1 Legend:** The table summarizes the 16 post-PrEP seroconverters by visit at which HIV infection was first detected, the history of PrEP use after PrEP initiation based on visit attendance and acceptance of PrEP refills, and laboratory data at the first post-seroconversion visit, including HIV-1 viral load, tenofovir diphosphate (TFV-DP) levels in dried blood spots (DBS) or plasma tenofovir (TFV) level, if DBS was not obtained, and resistance mutations based on partial RT gene sequencing. The mutations associated with tenofovir or emtricitabine are bolded. M= study visit after PrEP initiation. BLQ= below limit of quantitation.
